# Technical errors and complications in orthopaedic trauma surgery

**DOI:** 10.1007/s00402-015-2377-5

**Published:** 2015-12-21

**Authors:** M. A. Meeuwis, M. A. C. de Jongh, J. A. Roukema, F. H. W. M. van der Heijden, M. H. J. Verhofstad

**Affiliations:** Department of Surgery, St. Elisabeth Hospital, P.O. Box 90151, 5000 LC Tilburg, The Netherlands; Trauma Centre Brabant, St. Elisabeth Hospital, P.O. Box 90151, 5000 LC Tilburg, The Netherlands; Department of Surgery-Traumatology, Erasmus MC, University Medical Center Rotterdam, P.O. Box 2040, 3000 CA Rotterdam, The Netherlands

**Keywords:** Fractures, Surgical error, Complications

## Abstract

**Introduction:**

Adverse events and associated morbidity and subsequent costs receive increasing attention in clinical practice and research. As opposed to complications, errors are not described or analysed in literature on fracture surgery. The aim of this study was to provide a description of errors and complications in relation to fracture surgery, as well as the circumstances in which they occur, for example urgency, type of surgeon, and type of fracture.

**Methods:**

All errors and complications were recorded prospectively in our hospital’s complication registry, which forms an integral part of the electronic medical patient file. All recorded errors and complications in the complication registry linked to fracture surgery between 1 January, 2000 and 31 December, 2010 were analysed.

**Results:**

During the study period 4310 osteosynthesis procedures were performed. In 78 (1.8 %) procedures an error in osteosynthesis was registered. The number of procedures in which an error occurred was significantly lower (OR = 0.53; *p* = 0.007) when an orthopaedic trauma surgeon was part of the operating team. Of all 3758 patients who were admitted to the surgical ward for osteosynthesis, 745 (19.8 %) had one or more postoperative complications registered. There was no significant difference in the number of postoperative complications after osteosynthesis procedures in which an orthopaedic trauma surgeon was present or absent (16.7 vs. 19.1 %; *p* = 0.088; OR 0.85).

**Discussion:**

In the present study the true error rate after osteosynthesis may have been higher than the rate found. Errors that had no significant consequence may be especially susceptible to underreporting.

**Conclusion:**

The present study suggests that an osteosynthesis procedure performed by or actively assisted by an orthopaedic trauma surgeon decreases the probability of an error in osteosynthesis. Apart from errors in osteosynthesis, the involvement of an orthopaedic trauma surgeon did not lead to a significant reduction in the number of postoperative complications.

## Introduction

Adverse events result in morbidity and costs. The estimated direct medical costs attributable to adverse events during hospital admissions in the Netherlands in 2004 were more than € 355 million, about 2.4 % of the € 14.5 billion national hospital health care budget per year [1].

Adverse events can result from complications and errors. The relationship between an error and a complication is a causal one, although not all errors caused by caregivers necessarily lead to a complication for the patient. On the other hand, complications are not only caused by errors, but may also be due to the disease itself. Both errors and complications can result in no impairment, temporary impairment, or permanent impairment for the patient, which in turn might need additional treatment.

A previous study on errors in surgery showed a 6.1 % error rate in more than 12,000 patients admitted to surgical wards (including trauma ward); 16.8 % of patients developed one or more complications [2]. The error rate for patients admitted for trauma surgery was even higher (8.7 %).

In the literature, errors and complications that arise during surgery have been linked to a large variety of organisational and human factors, among others lack of surgeon specialisation [3, 4], surgical residents and trainees [5], low hospital volume [6, 7], conditions of increased patient complexity or systems failure [8], communication breakdowns [8, 9], fatigue [10], and time of day [11]. In the field of fracture surgery the relationship between complications and surgeon experience has been described for several procedures [12–14]. However, potential underlying errors are not yet further described or analysed.

Operative and nonoperative treatment of musculoskeletal injuries in the Netherlands is traditionally performed by surgeons with a general surgical background. Only 20 % of fractures are treated by surgeons with a general orthopaedic training, whose main workload consists of joint replacement and other elective musculoskeletal surgery.

In the last two decades, differentiation within the specialty of general surgery gradually evolved. This led to surgeons with a specific profile (i.e. gastro-intestinal, vascular, oncologic and trauma surgery) that still work within one group. Due to concentration of (trauma) patients in specific hospitals, a gradual change in case mix and work load developed. Between 2000 and 2010 many surgical groups in the Netherlands organised a 24/7 coverage with dedicated surgeons for all subspecialties. Nowadays, Dutch trauma surgeons treat both soft tissue injuries of thorax, abdomen and limbs (comparable to the anglo-saxon trauma surgeon), as well as up to 80 % of all fractures (comparable to the anglo-saxon orthopaedic trauma surgeon) [15]. Therefore, in this article a Dutch trauma surgeon will be referred to as an orthopaedic trauma surgeon.

The aim of this study was to describe all registered errors and complications in relation to fracture surgery in a level 1 trauma centre from 2000 to 2010. Furthermore, the circumstances in which they occur were analysed. We hypothesized that the number of errors and complications would drop as a result of the increasing differentiation (24/7 coverage with dedicated orthopaedic trauma surgeons).

## Methods

### Definitions

A medical error is defined as an act of omission or commission in planning or execution that contributes or could contribute to an unintended result [16]. A complication was defined according to the Association of Surgeons of the Netherlands as a condition or event, unfavourable to the patient’s health, causing irreversible damage or requiring a change in therapeutic policy [17]. An error has the potential to cause a complication. Both are considered to be preventable when there is a failure to follow accepted practice at the individual or system level. The definitions used were accepted by the entire surgical staff, and were used when documenting errors and complications.

### Registration method

This study was conducted in a secondary referral hospital and level 1 trauma centre, with a capacity of 673 beds. The surgical department consisted of 12–15 surgical residents, 8–10 consultant surgeons and 3–4 junior staff surgeons.

At the beginning of 1995 an electronic medical record was introduced in the hospital. The software used for the electronic medical record was an Oracle^®^ Forms (Redwood City, CA, USA) application with an Oracle database as back-end, which was developed by the hospital itself.

The electronic medical record has an integrated system for classifying complications developed by the Trauma Registry of the American College of Surgeons (TRACS which is further described elsewhere [18]). The TRACS system was originally developed as a complication list to record morbidity in trauma populations [19]. The list explicitly defines complications and uses four-digit codes. An advantage of the system is that it also allows registration of medical errors by specific codes [20, 21]. In the hospital, this integrated TRACS system allows physicians to register errors and complications in the operating room, in the wards, or in the outpatient clinic, regardless of patient outcome.

All events recorded are discussed during the daily surgical conference before final storage in the database. Only events judged by consensus to meet the definitions mentioned above were recorded in the complication registry. Furthermore, all procedures performed were analysed during the daily surgical conference along with the radiographs from the procedure and postoperative period.

### Data acquisition

In the present study, all osteosynthesis procedures performed between January 1, 2000 and December 31, 2010 were collected by performing a search in the Electronic Medical Record (EMR) database. For all patients operated during this study period, any error or complication due to the osteosynthesis procedure until discharge from outpatient follow-up was registered in the TRACS system.

The data of all osteosynthesis procedures performed during the study period were collected from the operating room database. This included fracture location, patient’s age, surgeon’s specialisation, presence of an orthopaedic trauma surgeon, elective or emergency setting, starting time, duration of surgery and need for a reoperation.

During the study period no osteosynthesis procedure was performed by residents without the supervision of a surgeon, independent of the field of interest of this surgeon. In this study, only surgeons who completed the full training (general training plus trauma differentiation) and received their certification by the Dutch Society of Trauma Surgery were regarded as orthopaedic trauma surgeon. During the study period the average time of experience per orthopaedic trauma surgeon was about 5–10 years after completion of their training. The involvement of an orthopaedic trauma surgeon in this study means the surgeon has scrubbed in and played an active role in the osteosynthesis procedure.

The EMR also provides a fracture template that can be completed by the surgeon after the procedure. It contains a fracture classification (Müller AO Classification of Fractures [22]) and a description of the soft tissue injury (Gustilo-Anderson Classification [23]), the degree of contamination (Surgical wound classification by the National Academy of Sciences [24, 25]) and the type of osteosynthesis performed.

Osteosynthesis procedures that are scheduled, at least 24 h ahead, are labelled as elective (non urgent) procedures. Emergency procedures are defined as procedures performed within 24 h after injury presentation, with a subcategory of procedures that are performed within 2 h.

The starting times of the procedures are divided into office hours (between 7:30 a.m. and 5:00 p.m.) and outside office hours (between 5:00 p.m. and 7:30 a.m., including Saturday and Sunday).

All errors in osteosynthesis were reviewed and subdivided in the following subcategories: inadequate fracture reduction, use of wrong implant, wrong length of implant, incorrect implant positioning, incorrect use of implant, or error in surgical approach. A certified orthopaedic trauma surgeon determined the fracture type using the Müller AO Classification of Fractures on the available conventional radiographic recordings [22].

### Statistical analysis

Descriptive analysis was performed in order to compare the circumstances (operation variables and patient variables) between procedures in which an error occurred and procedures in which no error occurred. The same was done for procedures followed by a complication and procedures not followed by a complication.

The characteristics of procedures in which an error occurred and procedures in which no error occurred were compared. Similarly, procedures that were followed by a complication were compared with those without complications. Finally, the rate of errors and complications linked to osteosynthesis procedures performed before and after July 1, 2009 were compared. From that date onwards, the involvement of a dedicated orthopaedic trauma surgeon became a requirement for conducting an osteosynthesis procedure. Pearson’s Chi squared tests were used in order to compare categorical variables. Odds ratios were calculated for categorical independent variables. Independent Student’s *T* tests were used to compare parametric continuous data. Mann–Whitney *U* tests were used to compare nonparametric continuous data. Differences were considered to be significant at a *p* level < 0.05.

Data were analysed using the Statistical Package for the Social Sciences (SPSS) version 16.0 (SPSS, Chicago, IL, USA).

## Results

During the study period 3758 patients were admitted to the surgical ward for osteosynthesis. In total 4310 osteosynthesis procedures were performed.

### Errors in osteosynthesis

In 78 (1.8 %) of all 4310 osteosynthesis procedures an error in osteosynthesis was registered. Relatively, most errors occurred in osteosynthesis procedures of the distal radius/ulna, proximal femur and malleolar segment, with an emphasis on complete intra-articular fractures of the radius, pertrochanteric fractures and trans- or suprasyndesmotic lesion of the malleolar segment (Table 1). Sixty-six of the 78 patients (84.6 %) were judged during the daily surgical conference to need a reoperation due to the error. Another eight patients were treated conservatively, in two patients the error was corrected during the primary operation and in two patients the implant (k-wires) was removed early.

**Table 1:** Errors in osteosynthesis by AO fracture classification

Bone or segment	Number of procedures with an error registered	Type	Errors
Distal radius/ulna	13 out of 486 (2.7 %)	(23-A) Extra articular fracture	3
		(23-B) Partial articular fracture of radius	2
		(23-C) Complete articular fracture of radius	8
Proximal femur	17 out of 654 (2.6 %)	(31-A) Trochanteric area	13
		(31-B) Neck	4
Malleolar segment	14 out of 669 (2.1 %)	(44-A) Infrasyndesmotic lesion	1
		(44-B) Transsyndesmotic fibular fracture	7
		(44-C) Suprasyndesmotic lesion	6

**Fig. 1:**
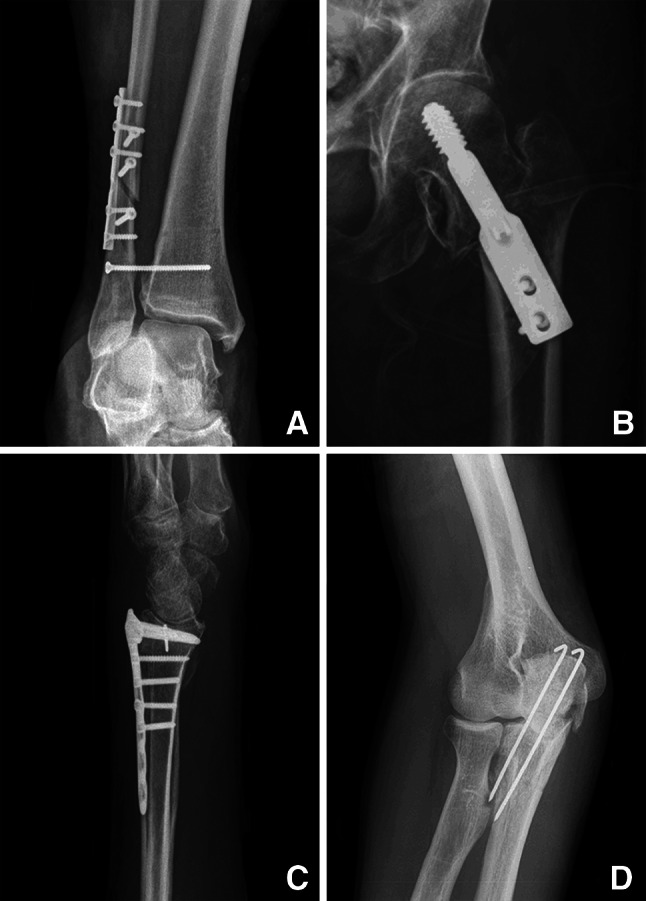
Errors in fracture surgery. **a** Inadequate fracture reduction. **b** Incorrect implant positioning. **c** Use of wrong implant (volar distal radial plate placed on dorsal side). **d** Wrong length of implant

In 13 patients the error could be assigned to more than one subcategory (Table 2). Both inadequate fracture reduction (Fig. 1a) and incorrect implant positioning (Fig. 1b) represented 30 % of all errors. The use of a wrong implant (Fig. 1c) occurred in 18 % followed by the use of an implant with an incorrect length (Fig. 1d) in 11 % of all cases. Incorrect implant positioning was the most common error in osteosynthesis of the distal radius/ulna and proximal femur. Inadequate fracture reduction was the most frequently occurring error in osteosynthesis of the malleolar segment.

**Table 2:** Errors in osteosynthesis by subcategory

Error subcategory	Number	%	Distal radius/ulna	Proximal femur	Malleolar segment
Inadequate fracture reduction	28	30	3	7	9
Incorrect implant positioning	28	30	5	11	4
Use of wrong implant^a^	17	18	4	2	3
Wrong length of implant	10	11	2	2	0
Incorrect use of implant^b^	8	8	1	1	1
Incorrect surgical approach^c^	1	1	0	0	0
Total	92	100	15	23	17

^a^Incorrect implant type used, for example volar distal radial plate placed on dorsal side

^b^Incorrect usage of implant, for example the omission to engage the locking mechanism of a collum screw when using a trochanteric femur nail™

^c^Incorrect fracture approach resulting in nerve damage

The number of procedures in which an error was registered was significantly lower (OR = 0.53; *p* = 0.007) when an orthopaedic trauma surgeon was present (Table 3). Analyses per bone segment did not show this significant difference, partly due to the small numbers of errors registered per bone segment. However, the same trend was observed.

**Table 3:** Procedure characteristics; error versus no error

	All operations	Error	No error	Odds Ratio^+^	*p* value
Osteosynthesis procedures^a^	4310	78	4232		
Patients age^b^ (years)	47 (26–65)	52 (38–68)	47 (26–65)		0.040^++^
Orthopaedic trauma surgeon^a^					
Present	3314 (76.9)	50 (64.1)	3264 (77.1)	0.53 (0.3–0.8)	0.007^+++^
Absent	996 (23.1)	28 (35.9)	968 (22.9)	Reference	
Duration procedure^b^ (hours)	1:13 (0:43–1:30)	1:20 (0:45–1:32)	1:13 (0:43–1:30)		0.122^++^
Start of procedure^a^					
During office hours	2721 (63.1)	45 (57.7)	2676 (63.2)	0.79 (0.5–1.2)	0.315^+++^
Outside office hours	1589 (36.9)	33 (42.3)	1556 (36.8)	Reference	
Urgency of procedure^a^					
Elective	1832 (42.5)	27 (34.6)	1805 (42.7)	Reference	0.271^+++^
Emergency >2 h	2227 (51.7)	45 (57.7)	2182 (51.6)	1.4 (0.8–2.2)	
Emergency <2 h	207 (4.8)	6 (7.7)	201 (4.7)	2.0 (0.8–4.9)	
Missing	44 (1.0)	0 (0)	44 (1.0)		

Data are shown as ^a^ number (percentage) or ^b^ median (P_25_–P_75_)

^+^ Univariate logistic regression, ^++^ Mann–Whitney *U* test, ^+++^ Pearson Chi Squared test

The median age was significantly higher in patients in whom an error occurred (52 vs. 47 years; *p* = 0.040). Other variables such as duration of the procedure, start time of the procedure (during or outside office hours) or setting (elective vs. emergency) did not differ between the procedures in which an error was recorded or not.

### Complications

One or more postoperative complications were registered in 745 of all 3758 operated patients (19.8 %) (Table 4). This was excluding errors in osteosynthesis. The most common complications were wound infections in 156 patients (4.2 %) and loss of reduction or fixation in 138 patients (3.7 %). A non-union was identified in 39 patients (1.0 %).

**Table 4:** Complications in relation to osteosynthesis surgery

	Total	Percentages^+^
Number of patients	3758	
Number of osteosynthesis procedures	4310	
Number of complications registered	967	
Number of patients with ≥ 1 complication registered	745	19.8
*Type of complication*		
Wound infection	156	4.2
Loss of reduction or fixation	138	3.7
Urinary retention	64	1.7
Haematoma/bleeding	49	1.3
Pneumonia	41	1.1
Urinary tract infection	40	1.1
Non-union^a^	39	1.0
Neurapraxia	38	1.0
Wound dehiscence	33	0.9
Pressure ulcus	24	0.6
Delirium	16	0.4
Other^b^	329	8.8

^+^ Percentage of all patients

^a^The definition used for a non-union is the failure to show any progressive change in bone healing after 6 months on radiographics

^b^Included but not limited to deep vein thrombosis, compartment syndrome and heart failure

There was no significant difference in the number of postoperative complications after procedures in which an orthopaedic trauma surgeon was present or absent (16.7 vs. 19.1 %; OR = 0.85; *p* = 0.088) (Table 5). Likewise, no significant difference was found if the complications were analysed separately.

**Table 5:** Procedure characteristics; postoperative complication versus no complication

	All operations	Complication	No complication	Odds ratio^+^	*p* value
Osteosynthesis procedures^a^	4310	745	3565		
Patients age^b^ (years)	47 (26–65)	55 (37–77)	45 (25–62)		<0.001^++^
Orthopaedic trauma surgeon^a^					
Present	3314 (76.9)	555 (74.4)	2759 (77.5)	0.85 (0.7–1.0)	0.088^+++^
Absent	996 (23.1)	190 (25.6)	806 (22.5)	Reference	
Duration procedure^b^ (hours)	1:13 (0:43–1:30)	1:28 (0:50–1:50)	1:10 (0:41–1:30)		<0.001^++^
Start of procedure^a^					
During office hours	2721 (63.1)	451 (60.5)	2270 (63.7)	0.90 (0.8–1.0)	0.106^+++^
Outside office hours	1589 (36.9)	294 (39.5)	1295 (36.3)	Reference	
Urgency of procedure^a^					
Elective	1832 (42.5)	269 (36.1)	1563 (43.8)	Reference	0.001^+++^
Emergency >2 h	2227 (51.7)	422 (56.6)	1805 (50.6)	1.4 (1.1–1.6)	
Emergency <2 h	207 (4.8)	46 (6.2)	161 (4.5)	1.7 (1.2–2.4)	
Missing	44 (1.0)	8 (1.1)	36 (1.0)		

Data are shown as ^a^ number (percentage) or ^b^ median (P_25_–P_75_)

^+^ Univariate Logistic Regression, ^++^ Mann–Whitney *U* test, ^+++^ Pearson Chi Squared test

The age of patients was significantly higher (55 vs. 45 years; *p* < 0.001) in the group of procedures followed by a complication. In addition, these procedures followed by a complication had a longer duration of 18 min (*p* < 0.001) and were more often performed in an emergency setting (*p* = 0.001).

### Severity of cases

A total of 1325 fracture templates were completed during the study period. Of these, 942 (71.1 %) concerned a procedure in which an orthopaedic trauma surgeon was involved. Analyses showed that complex fractures were more often operated when an orthopaedic trauma surgeon was involved, and concerned a type C fracture more often (OR 1.9; *p* < 0.001) (Table 6). There was no relation found between the involvement of an orthopaedic trauma surgeon and the degree of soft tissue injury or contamination.

**Table 6:** Severity of cases; orthopaedic surgeon present versus not present

	Orthopaedic trauma surgeon				
	All operations	Present	Not present	Odds ratio	*p* value
Completed fracture templates^a^	1325	942 (71.1)	383 (28.9)		
AO classification fracture type^b^					
A	611 (46.1)	417 (44.3)	194 (50.7)	Reference	<0.001^+^
B	404 (30.5)	276 (29.3)	128 (33.4)	1.0 (0.8–1.3)	
C	310 (23.4)	249 (26.4)	61 (15.9)	1.9 (1.4–2.6)	
Soft tissue injury (Gustilo–Anderson classification)					
Closed	1227 (92.6)	879 (93.3)	348 (90.9)	Reference	0.162^+^
Type I	55 (4.2)	39 (4.1)	16 (4.2)	1.0 (0.5–1.8)	
Type II	24 (1.8)	13 (1.4)	11 (2.9)	0.5 (0.2–1.1)	
Type III	19 (1.4)	11 (1.2)	8 (2.1)	0.5 (0.2–1.4)	
Surgical degree of contamination					
Clean	1197 (90.3)	854 (90.7)	343 (89.6)	Reference	0.380^+^
Contaminated	109 (8.2)	75 (8.0)	34 (8.9)	0.9 (0.6–1.4)	
Dirty	19 (1.4)	13 (1.4)	6 (1.6)	0.9 (0.3–2.3)	

Data are shown as ^a^ number (percentage). Odds ratio’s are shown with 95 % confidence interval

^+^ Pearson Chi Squared test

### Increasing differentiation

In the summer of 2009, the surgical department changed its policy regarding fracture surgery. The involvement of a dedicated orthopaedic trauma surgeon became a requirement for conducting an osteosynthesis procedure. Also, an orthopaedic trauma surgeon had to be available 24/7. Analyses comparing 1.5 years before and after this policy change (January 1, 2008 until June 30, 2009 vs. July 1, 2009 until December 31, 2010) showed no difference in the rate of errors (2.3 vs. 2.0 %; *p* = 0.731) or complications (10.4 vs. 11.3 %; *p* = 0.630).

## Discussion

In 78 (1.8 %) of all 4310 osteosynthesis procedures an error was registered. Sixty-six out of these 78 patients (84.6 %) needed revision surgery due to the error. Of all 3758 operated patients 745 (19.8 %) had one or more postoperative complications registered. The number of procedures with an error was significantly lower when an orthopaedic trauma surgeon was involved. Apart from errors in osteosynthesis, the involvement of an orthopaedic trauma surgeon did not affect the postoperative complication rate.

Despite the prospective registration of errors and complications this was a retrospective study with all its limitations. First of all, only variables that were registered routinely in the hospital database could be used in the analyses. Secondly, because of retrospective data collection it was not possible to determine whether errors, and in particular complications, were related to treatment or injury. For example, the significantly higher complication rate after emergency procedures (Table 5) could be related to the presence of additional injuries or comorbidities.

In the present study the true rate of error in osteosynthesis may have been higher than reported. Errors that had no significant consequences may be especially susceptible to underreporting, which may explain the high percentage of reoperations following an error (84.6 %) in the present study. Platz and Hyman [26] showed that surgeons fail to register approximately 13 % of all intraoperative complications. This corresponds with previous studies from our group that showed that the proportion of complications and errors captured by the prospective registry used in the present study was fairly high (73 and 90 %, respectively [27, 28]). The complication rate of 19.8 % in the present study is comparable to the rate found in a study conducted earlier by our group on complications and errors in surgery [2]. Due to the absence of the literature on error rates in osteosynthesis, the error rate of 1.8 % cannot be compared.

There are no indications that a difference exists in the accuracy in which residents, general surgeons or orthopaedic trauma surgeons register errors. All procedures performed are analysed blame free during the daily surgical conference along with the radiographs made during and after the procedure.

The age of patients was significantly higher in the group of procedures with an error (52 vs. 47 years; *p* = 0.040) or followed by a complication (55 vs. 45 years; *p* < 0.001). These results can perhaps partly be explained by an increase in osteoporotic bone at an older age. Osteoporotic fractures may be more complex with more extensive damage of cortical and cancellous bone, making it more difficult to achieve an adequate fracture reduction. Such fractures may also require a more extensive or other method of osteosynthesis. On the other hand, osteoporotic bone could also increase the risk of loss of reduction or fixation postoperatively, increasing the risk of complications. Nevertheless, a systematic review by Goldhahn et al. [29] could not prove a significant influence of osteoporosis on fracture fixation and complications.

The present study endorses the assumption that dedicated expertise improves quality of surgical care. The involvement of an orthopaedic trauma surgeon during the procedure seems to decrease the probability of an error in osteosynthesis. However, the involvement of an orthopaedic trauma surgeon does not appear to lead to a significant difference in the overall rate of postoperative complications, nor in the rate of wound infections, haematomas and loss of reduction or fixation separately. In this study, reoperations were performed in a small timeframe after the identification of an error in osteosynthesis. Patients were reoperated before the error could lead to other postoperative complications. Therefore, the fast majority of postoperative complications registered in this study (including loss of reduction or fixation and non-union) were not related to the identified errors. This may explain why the involvement of an orthopaedic trauma surgeon could lead to fewer errors in osteosynthesis without showing a significant reduction in postoperative complication rate. Perhaps, a larger study population could still have led to a significant difference in the overall rate of complications.

This also raises the question whether further differentiation of surgical expertise in orthopaedic trauma surgery alone, as seen in large trauma centres in the United States of America, is profitable. The number needed to treat is likely to grow and the benefits are getting smaller.

No difference in error or complication rate was found between the period before and after July 1, 2009 (January 1, 2008 until June 30, 2009 vs. July 1, 2009 until December 31, 2010). In this study, we decided to compare the same time window before and after the start date because error and complication rates show a fluctuation over time. This fluctuation is probably the result of a varying awareness and dedication over time together with changes in composition of the surgical staff. The change in policy by the surgical department to require involvement of a dedicated orthopaedic trauma surgeon for conducting an osteosynthesis procedure is reinforced by the result showing less errors in the group of osteosynthesis procedures performed with an orthopaedic trauma surgeon present. The present data did not confirm our hypothesis that the rate of errors and complications would drop as a result of increasing differentiation with 24/7 coverage by orthopaedic trauma surgeons. This might be a result of natural fluctuation of error and complication rates, an increasing level of quality due to differentiation along with higher demands, or simply an underpowered analysis as a consequence of the relatively low number of osteosynthesis procedures in the group after the intervention. Future analysis on larger data sets may be able to detect a changing trend in error and complication rates due to increasing differentiation.

## Conclusion

Errors in osteosynthesis seem to occur predominantly in complex fractures, which require an extensive procedure. The most common errors in osteosynthesis are inadequate fracture reduction and incorrect implant positioning. The present study suggests that an active role of an orthopaedic trauma surgeon during the procedure decreases the probability of an error in osteosynthesis.

Postoperative complications, not related to errors in osteosynthesis, are more prevalent after procedures performed in emergency settings, performed in older patients and with a longer duration. The involvement of an orthopaedic trauma surgeon during osteosynthesis procedures did not lead to a significant reduction in postoperative complications.
